# Hospital and Institutional News

**Published:** 1919-12-13

**Authors:** 


### HOSPITAL AND INSTITUTIONAL NEWS.

### THE THOMAS VICARY LECTURE, 1919.

Much historic interest is associated with the institution of this lectureship by the Royal College of Surgeons. In 1646, when both barbers and surgeons were associated in one corporate body, lectures were delivered, and their expenses were defrayed by a Mr. Edward Arris, and later by Mr. John Gale, who left to the Company an annuity of some £16 per annum for one anatomy lecture in every year to be called "The Gale Anatomy." In 1745 an Act of Parliament was passed dissolving the union between the barbers and surgeons, and the "Gale" and "Arris" lectureships were apportioned to the latter body. These lectures remain to the present time as a memorial of the ancient association between the barbers and the surgeons. Early in the present year, the Court of the Company of Barbers of London, expressed a desire to found a lectureship in the Royal College of Surgeons, on the history of Anatomy and Surgery, one lecture being given yearly for a term of five years, and to be called the "Thomas Vicary Lecture." Thus after an interval of many years, an association in learning has been established between the barbers and the surgeons. The first lecture was delivered by Sir John Tweedy on "The Surgical Tradition," many extracts of which will be quoted in another article in these pages. Sir John Tweedy was an ideal choice for the first lecture, if only for the fact that he is both a Past-President of the Royal College of Surgeons and a Past-Master of the Barbers' Company in London.

### HOSPITAL FINANCE IN GLASGOW.

In our issue of the 6th inst., page 204, by a printer's error the deficit of the Western Infirmary, Glasgow, was stated to be £117,853 in 1917 instead of £17,853. We are obliged to the treasurer and the secretary for calling our attention to this misprint.

### AN INTERESTING EXHIBIT.

During December and January at the Museum of the Royal College of Surgeons, Lincoln's Inn Fields, will be exhibited a case of surgical instruments which was the property of Dr. David Livingstone, and was carried with his body from the heart of Africa to the coast by his faithful native carriers in 1873-4. The case is almost complete with its equipment of instruments belonging to the surgery of bygone days and its authenticity is well attested by the manuscript which runs: "Case of surgical instruments, the property of the late Dr. David Livingstone, the African explorer, and presented to me by Dr. Oswell Livingstone, the traveller's only surviving son. This case was bought in 1865 by Dr. Livingstone of Messrs Hilliard, of Glasgow, who made and filled it to his order. It was used by him in his travels in Africa and was sent back, along with his other property, from that country in 1874 when the doctor's remains were brought to England. I hereby certify to the correctness of the above statement and the card attached was my father's visiting card (Signed) W. Oswell Livingstone, M.B., C.M.—W. M. Dunlop, October 15, 1885." The explanation is that the case had been acquired from Dr. Dunlop, a fellow student of the explorer's son in Glasgow, by Dr. Chidall, and will ultimately be presented to the London Missionary Society, which originally sent Livingstone to Africa.

### THE MEASLES ORDER.

The Notification Order now withdrawn has been most useful in giving medical officers relatively exact knowledge of the prevalence and distribution of measles during an epidemic period, but apart from that, it is doubtful whether it has aided greatly in directly checking the spread of the disease. It is interesting to note that the change does not affect Birmingham, where the local Act of 1914 makes it necessary for parents to report the occurrence of a case of measles, german measles, whooping-cough, or chicken-pox, when a child from the infected house is attending school. But the medical officer of this district does not himself, according to the local press, find much preventive value in the notification of measles. The proposals of the Health Ministry in relation to future practical methods are now well known. A very great amount of welfare and visitation work is thereby called for, but it must also be remembered by those who are inclined to add to the outcry against the country's attitude towards this disease that the Ministry are prepared to pay one-half of the cost to any municipality which undertakes this work.

### INTERNATIONAL RESEARCH.

It is good to learn on the authority of the *Times* that there will be a research department founded in London in connection with the new American hospital. It is a hope more than likely to be fulfilled that this development will encourage workers from America to take up investigations in this country, and also to place at the disposal of British workers such facilities as they may stand in need of. In this way a further link betweeen the two nations will be forged, and an invaluable impetus given to research work, international in character.

### VENEREAL NOTIFICATION.

It is decided that, owing to the great increase in the number of venereal cases now attending the London hospitals under the joint arrangements with the local authorities—an increase of more than 60 per cent. above the figure for 1918—it will be necessary to provide additional staff, equipment and accommodation. The Public Health Committee have communicated the facts to the Minister of Health, and will recommend the Council to send a communication to the authorities of the London hospitals drawing attention to the desirability of adequate provision for the present increase, and informing them that when satisfactory arrangements have been made, the question of additional cost will receive the sympathetic consideration of the Council in connection with grants for the year 1920-21.

### THE FUTURE OF CONTRACT PRACTICE.

There is considerable interest for the general practitioner to be derived from the fact that at the recent meetings, held under the auspices of the British Medical Association and the Medico-Political Union respectively, a substantial agreement was arrived at both with regard to the size of the capitation fee for which insurance practitioners are prepared to render efficient and willing service and to the effect on the goodwill of an insurance practice of the new clause in the draft of the Medical Benefit Regulations for 1920. There is, however, some difference in the two suggested methods of bringing pressure to bear on the Ministry of Health should this authority find itself unable to sanction what one way assume to be the considered demand of at least a considerable majority of the profession. On the whole, one would perhaps not favour at such a time as the present the direct policy expressed by the members of the Union, which amounts to a precise demand and ultimatum to the Minister of Health. Already through a leading article of the *National Insurance Gazette* and by letters from Dr. Addison himself it is abundantly clear that Ministerial opposition will undoubtedly be met. As we go to press a deputation from the Insurance Acts Committees has arranged to meet the Minister, and we await with no little interest the result of his discussion.

### UNIDENTIFIED SOLDIERS.

Every now and again the secretary of a voluntary hospital receives a pathetic letter of inquiry, generally enclosing a photograph, from the relatives of a soldier who was lost in the war, a forlorn hope still remaining in their minds that he may be suffering from loss of memory or other infirmity and has not yet been identified. Answering a question in the House of Commons addressed to the Secretary of State for War, Mr. Forster stated that he understands that there are three cases of soldiers whose identity it has not been possible to establish owing to mental trouble, all of whom are in this country, and everything possible is being done to clear up these cases. Would it not be as well to make public where these cases may be heard of in order that the sad suspense that is evidently still felt by some who have lost their relatives, and have found no trace, may be kindly and definitely terminated?

### DOCTORS' INCREASED FEES.

Acting on the recommendation of the Council of the B.M.A., local branches all over the country are advising the raising of medical fees in compensation for the depreciation in value of the pound. This week the following resolution was passed by the Marylebone branch, which, incidentally, includes Harley Street. "In view of the altered value of money, this meeting of the Marylebone Division of the B.M.A. advises members of the profession practising in the borough . . . to raise their professional fees by at least 50 per cent. on rate prevailing before the war."

### AMATEUR ANÆSTHETICS.

An inquiry was concluded at the City Coroner's Court on December 5 into the death of Robert Warner. Dr. Waldo said that a medical correspondent had written to the *Times* saying it seemed a pity that more scientific attention had not been devoted to the subject. If, he added, the Medical Research Committee would take the matter up a service to the community would be performed. His earlier and full recommendations are published on a later page of this issue, and it is satisfactory to learn that the jury returned a verdict in accordance with the medical evidence, and added a rider that no general or local anæsthetic should be administered by any but a duly qualified medical man, except in most exceptional circumstances, and that the Medical Research Committee and the Ministry of Health be requested to inquire into all matters connected with anæsthetics given for operations. The jury unanimously agreed with the coroner that legislation with regard to the administration of anæsthetics was urgently needed.

### THE CONDITION OF BANBRIDGE INFIRMARY.

Mr. Fitzpatrick, Local Government Board Inspector, recently inspected the workhouse of the Banbridge Board of Guardians, Ireland, and severely criticised, not for the first time, the conditions obtaining there. He complained that the inmates were allowed to use their dormitories during the day, and that the present sanitary conditions of the infirmary and fever hospital are "a disgrace to any institution." Nothing, he said, has been changed since his previous report, the woodwork itself is "perishing for want of paint." The matter has been deferred by the Guardians to a meeting for which a whip has been sent to the Guardians. Now that the Poor-law system is threatened, it is well to contrast such a case as that of Banbridge with the defence that is being offered against any change. If this is objected to because the institution is in Ireland, the reply is that what the Irish Poor-law system allows to-day was common in England yesterday. Is there any authority except the Poor-law which allows such a state of affairs?

### THE BISHOP'S HUMOUR.

The Bishop of Worcester has lately been active on behalf of Worcester Infirmary, which, as our readers know, has not yet pulled itself out of the financial slough into which it fell of recent years. A modest sum of £10,000 is in process of collection. In supporting this the Bishop declared that all classes were now necessary to the support of the voluntary hospitals. He and his entire household expected to emigrate there shortly, because the old boiler at the castle, which had broken down, could not be replaced, and in the absence of warmth he foresaw serious illness in his family. For this reason, if for no other, he thought that the infirmary should be helped. We agree. For one good reason why every class should support the voluntary hospitals is that every class now uses them. They stand between the Poor-law infirmary, which they have done much to improve, and the nursing-home, which they have done much to expose. They are, in fact, the one sound part of our hospital system. Democracy means the creation of one vast middle class, which can provide for itself except in times of illness or emergency. At this time the hospital comes to the rescue, and the Bishop of Worcester's humour is a happy reminder of this fact.

### THE IRISH OUTRAGES.

One of the Dublin hospitals which periodically has to treat the victims of Irish outrages is Steevens'. This lately received a hearty tribute from the Inspector-General of the Royal Irish Constabulary, Sir J. A. Byrne. He had promised to address a meeting of the supporters of the hospital's Linen League, but had been unable to prepare a speech because he had been called away in connection with the cowardly and senseless murder of one of his men. Steevens' Hospital continually gave splendid treatment to members of the Force. In its wards the men were invariably cheerful and comfortable. How significant to us on this side of the Irish Channel are these words! The fact that Steevens', and no doubt other Dublin hospitals, are thanked for their "continual treatment" of the Royal Irish Constabulary shows the risks now run by these men, and the state of unrest which lies behind them.

### THE HEADQUARTERS OF THE WELSH PRIORY.

Thanks to Lord Bute's generous help the Welsh Priory of the Order of St. John of Jerusalem is to have its centre in Cardiff, on the site of the former Old Priory of Grey Friars. The Priory will be the centre, it is hoped, of all the vountary hospitals and nursing bodies in Wales, and through this co-ordination the Ministry of Health will have a single main body to deal with. It is prophesied also that by its help lucrative appointments for nurses with full certificates and university diplomas will become available.

### HOSPITAL TICKETS AND A POUND OF TEA.

It is rarely that an Irishman is able to congratulate his country on anything except historic grievances, and we therefore read with interest the address of Mr. R. J. Johnstone, F.R.C.S., on the Irish Medical Services. He told his Belfast audience that the Irish Dispensary Service was one of the biggest health services in existence. He believed it was unique, but since the eight hundred State-paid doctors employed by the Irish Boards of Guardians were at everybody's beck, since there was no statutory definition of poverty, many abuses had arisen. In several county districts guardians who were also grocers handed red dispensary tickets with the groceries to their customers. It was, he said, the existence of the dispensary system which made the application of the medical clauses of the Insurance Act unnecessary and unenforced in Ireland. The appointments of the dispensary doctors, however, were open to improvement, better prospects of promotion and retiring allowances should be provided. He advocated the appointment by a central authority of county medical officers of health. A State medical service would forbid people full choice of their doctors, and would increase officialdom in every direction.

### THE GUEST HOSPITAL, DUDLEY.

The most practical suggestion yet made to drag the Guest Hospital, Dudley, from the unfortunate position into which it has fallen has come from Sir Gilbert Claughton, who consented to preside at the recent annual meeting. He admitted the force of many of the criticisms made against the management, criticisms with which we have had to deal in the past, and suggested a round-table conference of those opposed and those interested in the present administration. This proposal happily coincides with a statement of one of the hospital's critics, the *Dudley Herald*, which has truly reminded the management that "nothing is more favoured by a generous public than a properly managed hospital." To restore confidence is the duty of the moment, and that can be done only by reforming the present administration.

### A FAR-REACHING APPEAL.

The institution known as the Eversfield Chest Hospital, at St. Leonards, is in need of £22,000 to redeem a mortgage. Where is it to seek support? The answer is, in Yorkshire; and, no doubt a very good answer too. At all events the acting secretary of the Eversfield Chest Hospital, Mr. E. S. Gambier, has sent a statement of the case to the *Yorkshire Post*. Whether Mr. Gambier is merely enforcing a connection between Yorkshire and St. Leonards does not matter. If he is not, his tactics are the more interesting. We lately had evidence of the value of advertisements in parish magazines. We doubt if the value of announcements and letters in provincial papers is enough recognised, and applaud experiments in this direction. If Mr. Gambier can tell us of the success of his policy we shall tell, for instance, Mr. Leonard Rea, of Cardiff, so that he may shame the local public by seeking support from, perhaps, the Scilly Isles or Salisbury Plain or the Grampians.

### THE AGED POOR AND IDEAL HOMES.

Lambeth Board of Guardians are asking the Ministry of Health for permission to close one of their workhouses in North Lambeth and their Home for the aged poor at Norwood. This is due to the great decline in the number of inmates. The workhouse, which is situated in Prince's Road, is one of the old barrack-like institutions, where stone-breaking and working a mill for the grinding of corn are the only tasks existing for a able-bodied man seeking relief. With regard to the aged poor, however, a different story can be told. The home was built for the special accommodation of old couples forced to seek institutional help in their declining years. It is an ideal home situated on the outskirts of the borough, and this is the chief reason why it has not appealed to those for whom it was opened. The bulk of the old folk have lived in North Lambeth, where no trees and grass are to be seen, where there is plenty of noise and bustle. They prefer to end their declining years amidst these uncongenial surroundings rather than in the quietude of Norwood. Hence their objection to the Home for the aged poor. Another point is that out there they are far removed from their friends and relatives. Accommodation was provided for 108 old folk, but only four married couples could be persuaded to go to Norwood. The ideal has gone, and the place is to be closed, because as Mr. Collins, one of the members, remarked, the old folk like to live where they can hear the barrel-organs and the street pianos rather than dwell amidst surroundings.

### THE SALARIES OF PHARMACISTS AT VOLUNTARY HOSPITALS.

A CORRESPONDENT with some experience of hospital pharmacy writes to the *Chemist and Druggist* complaining of the inadequate salary offered for the post of Assistant Pharmacist at Guy's Hospital—recently advertised at £150 per annum. It is to be remembered that in order to obtain the statutory qualification of "Pharmacist," a three years' curriculum is requisite, this comprises a college course of nine months, and, in addition, two years' practical training in a pharmacy (usually without pay, either in cash or by way of board and lodging). At Poor-law infirmaries and other public institutions, the pharmacist at present receives a substantial "War bonus" and in this respect is far better off financially than many Assistant Pharmacists at voluntary hospitals. Indeed, at several Poor-law infirmaries the total wages of a porter is at present £3 10s. per week, and it should require little argument to convince the governing authorities of voluntary hospitals that their pharmacists should be paid a salary more commensurate with the responsible work they are engaged in. The point is emphasised in regard to Guy's, that no board, lodging, or meals are provided, and also that, as compared with other skilled workers and even many unskilled, the salary is quite inadequate. Despite the handicap of lessened income and ever increasing expenses, it would appear that voluntary hospitals ought to bring the salaries of their Assistant Pharmacists up to a level approximating to those paid by retail pharmacists.

### HOSPITALS AND STATE MAINTENANCE.

EDMONTON Urban District Council have adopted a resolution, which they are asking other public authorities to support, that they consider the time more than ripe for the taking over of the voluntary hospitals by the State. They contend that these institutions are too valuable to be left to charity, seeing that it is impossible by past experience to raise sufficient funds by begging for their proper upkeep, maintenance, and administration; and therefore they hope that the whole of the hospitals of this country will be taken over by the State, and properly provided for at the earliest possible moment. This far-reaching and drastic resolution has been submitted to the consideration of several Metropolitan borough councils, but so far has not met with any support.

### MUNICIPAL HOSPITAL FOR WALTHAMSTOW.

THE Health Committee of the Walthamstow Urban District Council has prepared a scheme for taking over Brookfield Hospital—offered for sale for £5,500. The Committee reports that these premises can be adapted inexpensively for use as a hospital for children under five years of age: to serve as a crêche or day nursery; as a convalescent home for nursing mothers and for children under five; and for attending to the health of children of widowed, deserted, and unmarried mothers.

### WHO IS THE KING OF ISRAEL?

ALL interested in the encouragement of subscriptions from employers as well as from their men will like to see the reason assigned at West Bromwich for the bad example set by some of the big firms in regard to their contributions to the hospital. If, said Mr. Albert Leach, the chairman of the Saturday Committee, we can approach the works manager direct, there is no difficulty, but the way is baffled sometimes between us and him. It is much to know who is the person who decides these things that, on Biblical precedent, we may fight with him, and waste no attention upon others.

### THE WARRIORS' RETURN.

MR. J. H. JOHNSON, the Secretary of the Royal Westminster Ophthalmic Hospital, has resumed his duties as secretary of the hospital, after two years' active service in Palestine. On the lay side of hospital life most of the officers who were on military service at home and abroad have now resumed their civilian work. Amongst these may be mentioned Mr. A. G. Elliott of the London Hospital, Mr. Whitney of the Heart Hospital, Mr. George Watts of Victoria Park Consumption Hospital, Mr. H. W. Burleigh of Maida Vale Nervous Disease Hospital, and Mr. H. K. S. Druce of the Central London Ophthalmic Hospital. We understand that Mr. Darmady (better known as Mr. Stewart Johnson) of Great Ormond Street Hospital will not return to hospital work. Very sensibly most of these gentlemen, and others who held temporary commissions, are not retaining their military titles in civil life, and their services to their country are none the less appreciated. The decision is one that must be applauded, because there might arise a state of affairs similar to that existing in the United States after the Civil War, when, as was said by a humorist, one could not "throw a stick in a crowd without mowing down two lieutenant-colonels and a major."

### THIS WEEK'S DRUG MARKET.

SINCE the removal of the restrictions on dealings in quinine sulphate the price has advanced substantially, and the market is unsettled; in view of the large demand for quinine in malarial countries, it is probable that a high price will be established. In consequence of the rise in quicksilver, makers of mercurials have raised their quotations by sixpence per lb. for all the salts except corrosive sublimate. The value of opium is maintained, and makers of morphine are very busy. Camphor has again advanced in price, and the tendency is still in an upward direction. Sugar of milk tends upwards in price. The state of the American exchange naturally has the effect of pushing up the prices for drugs obtained from the United States, including some of the synthetic drugs. Ergot of rye still moves upwards in price. Menthol is again dearer. The values of ipecacuanha and senna are firmly maintained.

